# Financial incentives to improve glycemic control in African American adults with type 2 diabetes: a pilot randomized controlled trial

**DOI:** 10.1186/s12913-020-06029-0

**Published:** 2021-01-13

**Authors:** Leonard E. Egede, Jennifer A. Campbell, Rebekah J. Walker, Aprill Z. Dawson, Joni S. Williams

**Affiliations:** 1 Division of General Internal Medicine, Department of Medicine, Medical College of Wisconsin, 701 Watertown Plank Rd, Milwaukee, WI 53226-3596 USA; 2 Center for Advancing Population Science, Medical College of Wisconsin, Milwaukee, WI USA

**Keywords:** Financial incentives, Randomized controlled trial, Diabetes, African American, Glycemic control

## Abstract

**Background:**

Financial incentives is emerging as a viable strategy for improving clinical outcomes for adults with type 2 diabetes. However, there is limited data on optimal structure for financial incentives and whether financial incentives are effective in African Americans with type 2 diabetes. This pilot study evaluated impact of three financial incentive structures on glycemic control in this population.

**Methods:**

Sixty adults with type 2 diabetes were randomized to one of three financial incentive structures: 1) single incentive (Group 1) at 3 months for Hemoglobin A1c (HbA1c) reduction, 2) two-part equal incentive (Group 2) for home testing of glucose and HbA1c reduction at 3 months, and 3) three-part equal incentive (Group 3) for home testing, attendance of weekly telephone education classes and HbA1c reduction at 3 months. The primary outcome was HbA1c reduction within each group at 3 months post-randomization. Paired t-tests were used to test differences between baseline and 3-month HbA1c within each group.

**Results:**

The mean age for the sample was 57.9 years and 71.9% were women. Each incentive structure led to significant reductions in HbA1c at 3 months with the greatest reduction from baseline in the group with incentives for multiple components: Group 1 mean reduction = 1.25, Group 2 mean reduction = 1.73, Group 3 mean reduction = 1.74.

**Conclusion:**

Financial incentives led to significant reductions in HbA1c from baseline within each group. Incentives for multiple components led to the greatest reductions from baseline. Structured financial incentives that reward home monitoring, attendance of telephone education sessions, and lifestyle modification to lower HbA1c are viable options for glycemic control in African Americans with type 2 diabetes.

**Trial registration:**

Trial registration: NCT02722499. Registered 23 March 2016, url.

## Background

Diabetes affects 34.2 million people or 10.5% of the US population [1]. African Americans experience a disproportionate burden of diabetes through higher prevalence, poorer metabolic control, increased risk for complications, and higher rates of mortality compared to non-Hispanic Whites [1]. African Americans are also less likely to receive recommended diabetes quality of care measures, have lower engagement in self-management behaviors, and report lower receipt of diabetes self-management education compared to non-Hispanic Whites, lending to existing disparities in metabolic control and clinical outcomes for African Americans [1–11].

Cultural tailoring combined with high intensity behavioral interventions are shown to reduce the burden of diabetes for African Americans through improved metabolic control [12–15]. Specifically, components of effective lifestyle interventions for African Americans with diabetes includes targeting self-care behaviors that influence glycemic control through telephone delivered nurse coaching, telemonitoring, and skills training [16–22]. Evidence also shows that culturally tailoring telephone delivered diabetes education and skills interventions that emphasize self-care goal setting are effective in improving glycemic control among African Americans [5, 13]. Additionally, recent evidence drawn from behavioral economics research suggests that financial incentives as a component of lifestyle interventions for African Americans with diabetes may further reduce health disparities through targeting self-care behaviors and improving glycemic control [23].

Behavioral economics research has identified several decision-making patterns that contribute to poor adherence to medical regimens with delay discounting representing the extent to which consequences or outcomes decrease in their effectiveness to control behavior the longer the consequences or outcomes are delayed [24, 25]. For example, adults with diabetes who prefer smaller, immediate benefits (e.g. eating preferred foods or not being inconvenienced by exercising or taking medications) over larger, delayed benefits (e.g. better metabolic control, reduced risk of complications, or reduced risk of dying), are less likely to make healthy lifestyle decisions. Therefore, using financial incentives as an intervention component to improve diabetes outcomes through incentivizing self-management behavior may be effective in increasing self-management and improving outcomes [23, 26–28].

Preliminary evidence suggests that financial incentives can lead to improvements in glycemic control in African Americans with diabetes [23]. However, no large scale randomized clinical trial has examined whether financial incentives are effective in improving metabolic control in African Americans with diabetes and whether financial incentives augmented telephone-delivered diabetes education and skills training intervention will lead to greater improvements in metabolic control compared to usual care. The current study serves as a feasibility and preliminary efficacy trial of a structured financial incentive intervention on glycemic control in African American adults with type 2 diabetes.

The overarching aim of this study is to test the efficacy of three financial incentive structures in combination with technology intensified diabetes education and skills training intervention on blood pressure and quality of life in African Americans with type 2 diabetes. Sixty African Americans with type 2 diabetes were randomized to three groups with varying frequency of financial incentives: 1) High Frequency: financial incentives for weekly uploads plus average glucose, incentives for weekly attendance to educational sessions, and incentives at the end of the study for meeting HbA1c goals 2) Moderate Frequency: financial incentives for weekly uploads plus average glucose, and incentives at the end of the study for meeting HbA1c goals, and 3) Low Frequency: financial incentives at the end of the study for meeting HbA1c goals.

We hypothesized that structured financial incentives would be associated with significant reductions in glycemic control as measured by HbA1c within groups at 3 months post randomization. Given this was a pilot study, it was not powered to detect between group differences.

## Methods

### Aim, design, setting

This was a randomized trial with three study groups and a 3-month follow-up. Sixty (60) adult African Americans with diagnosed type 2 diabetes were randomized to one of three financial incentive structures and received 12 weeks of telephone-delivered diabetes education and skills training provided by a trained nurse educator. Institutional Review Board (IRB) approval was obtained for this study from the IRB at the Medical University of South Carolina in Charleston, South Carolina. Study participants were recruited from out-patient clinics and primary care clinics. This study is registered at clinicaltrials.gov (identifier: NCT02722499). This study adheres to CONSORT guidelines for reporting pilot and feasibility trials.

The inclusion criteria for the study included:

1. Age ≥ 21 years
2. Clinical diagnosis of T2DM and HbA1c ≥8% at the screening visit indicating poor control
3. Self-identified as African American (participants were asked if they self-identify as being Black/African American)
4. Taking an oral medication or insulin for diabetes to be able to assess medication adherence
5. Able to communicate in English
6. Access to a telephone (landline or cell phone) and/or ethernet for the study period; and
7. Willing to use the FORA monitoring system for 3 months.

The exclusion criteria for the study included was based on the ability of participants to engage in 12 weeks of education and subsequent follow up visits independently and therefore included:

1. Mental confusion on interview suggesting significant dementia
2. Participation in other diabetes clinical trials
3. Alcohol or drug abuse/dependency
4. Active psychosis or acute mental disorder
5. Life expectancy < 12 months.

A variety of recruitment methods were employed to identify and enroll interested study participants. The first involved use of an IRB approved recruitment flyer placed in outpatient clinics, exam rooms, and public areas such as cafeterias, waiting rooms, and information desks at the medical center. The second strategy included use of physician signed invitation letters mailed to patients with type 2 diabetes. Thirdly, healthcare providers referred patients to the study, and finally, clinic billing records were used to identify African American patients with type 2 diabetes. Patients who expressed interest in participating were scheduled for a screening visit where a venous sample of blood was collected and HbA1c lab analysis was used to assess eligibility. Once determined eligible, patients returned 1 week later to be enrolled and randomly assigned (1:1:1) to one of three study arms.

Participants were randomized to three groups with varying frequency of financial incentives: Group 1: Low Frequency Financial Incentives - patients received a reward for absolute percentage drops in HbA1c from baseline at 3-month follow-up, up to $300. Group 2: Moderate Frequency Financial Incentives - patients received a reward or uploading glucose measurements, and absolute percentage drops in HbA1c from baseline at 3-month follow-up, up to $300. Group 3: High Frequency Financial Incentive – patients received a reward for uploading glucose measurements, attending educational sessions, and absolute percentage drops in HbA1c from baseline at 3-month follow-up, up to $300. The study included 20 participants in each arm (total *n* = 60) to have 80% power to detect at least a 0.5 standardized effect within each intervention group (difference between baseline and 3-months HbA1c). An intraclass correlation coefficient of > 0.6 and 0.05 level of significance were assumed. The randomization sequence was stratified by HbA1c where one stratum included patients with HbA1c 8% - < 10%, and the 2nd stratum included patients with HbA1C > =10%. Implementation of random allocation sequence included the use of sequentially numbered sealed envelopes that were opened with participant at the time of randomization. Random allocation sequence was generated by a biostatistician. Research staff enrolled participants and were blinded to sequence, research nurses completed intervention assignment based on allocation sequence listed in sealed envelope.

### Intervention

All study participants were provided 1) telephone-delivered diabetes education and skills training for 12 weeks with a trained nurse educator, 2) a validated culturally tailored education booklet that provided local and culturally relevant resources for diabetes self-management [29], 3) patient activation through discussion with nurse educator regarding questions to ask health provider, 4) a FORA 2-in-1 telehealth device used to monitor blood glucose, and 5) one of three structured financial incentives.

Telephone-Delivered Diabetes Education and Skills Training: Participants received weekly telephone-delivered diabetes knowledge/information, patient activation, patient empowerment, and behavioral skills training delivered via telephone. The intervention was delivered by telephone once a week for 12 weeks with each session lasting approximately 30 min. The telephone was used as the mode of delivery based on prior evidence suggesting the effectiveness and high engagement based on patient preference of telephone delivered education [20].

Financial Incentives: The financial incentives component of the intervention was developed based upon the delay discounting and financial incentives literature [23, 26–28]. This part of the intervention was designed to: a) target multiple behaviors that contribute to glycemic control, b) be given at a frequency that is consistent with expected change in HbA1c, c) be large enough to motivate behavior change in the target population, d) reward more aggressive lowering of HbA1c to the target of < 7%, and e) reward maintenance of targeted behaviors as indicated by maintenance of HbA1c levels at each post-baseline assessment point. The three structured incentives included are shown in Table 1 below.

**Table 1:** Financial Incentive Structure

Group	Description	Incentive Structure
Group 1: Low Frequency Financial Incentive	Single incentive at 3-months for absolute percentage drop in HbA1c from baseline to 3-month follow-up, up to $300.	HbA1c Reduction: After 3 months, if their HbA1c has dropped 2% from baseline, or absolute HbA1c is 7%, they will receive a reward of $300. For a 1% drop, or an absolute HbA1c between 7 and 8 they will receive a reward of $150
Group 2: Moderate Frequency Financial Incentive	Single two-part incentive for uploading glucose measurements through home testing of glucose and absolute percentage drop in HbA1c from baseline to 3-month follow-up, up to $300.	Glucose Uploads: Each week participants in Group 2 can receive up to $10 for uploading glucose measurements and having good glucose control throughout the week. For each day they upload at least one glucose measurement, they will receive $1 (up to $7 at the end of the week). If they upload measurements every day of the week and their average glucose measurements at the end of the week are 150 or below, they will receive an additional $3 for up to $10 per week for 3-months. HbA1c Reduction: After 3 months, if their HbA1c has dropped 2% from baseline, or absolute HbA1c is 7%, they will receive a reward of $170. For a 1% drop, or an absolute HbA1c between 7 and 8 they will receive a reward of $85.
Group 3: High Frequency Financial Incentive	Single multiple component incentive for uploading glucose measurements using home testing, attending weekly phone educational sessions, and absolute percentage drop in HbA1c from baseline to 3-month follow-up, up to $300.	Glucose Uploads: Each week participants in Group 3 can receive up to $10 for uploading glucose measurements and having good glucose control throughout the week. For each day they upload at least one glucose measurement, they will receive $1 (up to $7 at the end of the week). If they upload measurements every day of the week and their average glucose measurements at the end of the week are 150 or below, they will receive an additional $3 for up to $10 per week for 3-months. Attendance of Educational Sessions: Educational sessions will last for 8 weeks, so they can receive up to $5 per week for 8 weeks. HbA1c Reduction: After 3 months, if their HbA1c has dropped 2% from baseline, or absolute HbA1c is 7%, they will receive a reward of $130, for a 1% drop, or an absolute HbA1c between 7 and 8 they will receive a reward of $65.

### Primary outcome

The primary clinical outcome of interest was HbA1c reduction within each arm at 3-months post randomization.

### Statistical analysis

Descriptive statistics including frequency, percent, and mean were calculated for the sample overall, and stratified by intervention group. Differences in demographic variables were investigated across the three intervention groups using oneway ANOVA for continuous variables and Fisher’s exact tests for categorical variables. Unadjusted means for the clinical outcome, HbA1c were calculated for baseline and 3-month follow-up for each intervention group. Change in HbA1c was also calculated for each individual and summarized by mean and standard deviation for each intervention group. Paired t-tests were used to assess differences in HbA1c from baseline to 3 months within each group. All analyses were performed using Stata version 15.0 (StataCorp, College Station, TX).

## Results

**Fig. 1:**
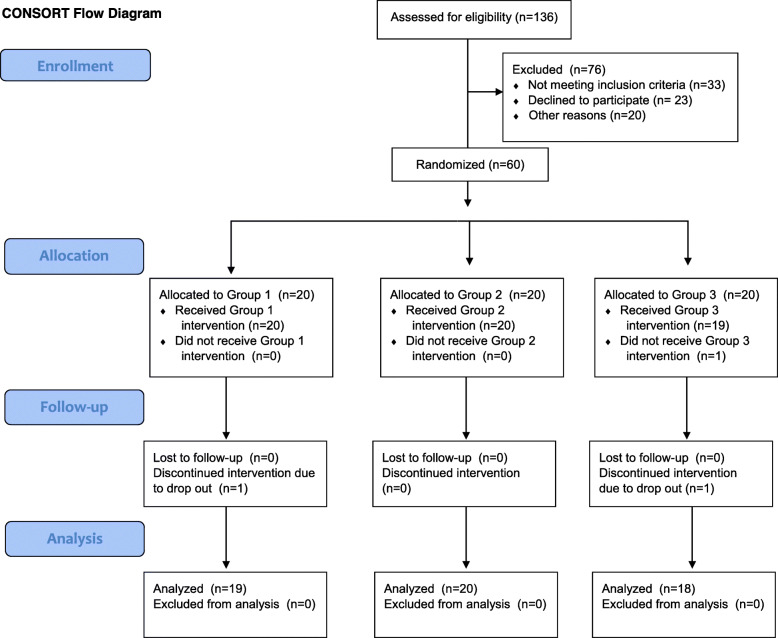
Consort Flow Diagram

Between March 1, 2016 and July 31, 2016, 60 participants were randomized to Group 1 Low Frequency Financial Incentive (*n* = 20), Group 2 Moderate Frequency Financial Incentive (*n* = 20), or Group 3 High Frequency Financial Incentive (*n* = 20). Figure 1 shows the CONSORT Diagram describing the flow of patients from eligibility to analysis. There was a total of 60 participants at baseline. One participant dropped out of study Group 1 prior to receiving the intervention. One participant dropped out of study Group 3 prior to receiving the intervention and one participant’s enrollment was discontinued prior to receiving the intervention from study Group 3 after disclosing a new diagnosis with the research nurse that would deem it impossible to continue in the research activities as outlined in the protocols inclusion criteria. Accounting for dropouts (*n* = 2) and discontinuation (*n* = 1), 57 participants out of 60 were included in the final analysis who had complete data. Table 2 provides demographics of the sample overall and by intervention arm. Mean age was 57.4 years, on average participants had been diagnosed with diabetes for 16.6 years, and 70.5% were female. There were no significant differences in demographics by intervention arm.

**Table 2:** Sample Demographics for Study Overall and By Intervention Arm

	Overall Sample	Group 1	Group 2	Group 3	***P***-value
	Mean (standard deviation or percent)				
Age	57.4 (11.4)	58.1 (14.1)	57.5 (8.8)	58.2 (9.6)	0.106
Duration of Diabetes	16.6 (10.8)	18.6 (13.4)	17.7 (9.2)	17.2 (11.4)	0.297
Yrs of Education	13.1 (2.8)	12.8 (1.7)	12.3 (1.8)	14.9 (2.6)	0.109
Sex					
Male	29.5%	25%	37.5%	37.5%	0.758
Female	70.5%	36.6%	29.3%	34.2%	
Marital Status					
Married	29.6%	37.5%	12.5%	50%	0.128
Not Married	70.4%	31.7%	39%	29.3%	
Income					
Less than $20,000	51.1%	40%	40%	20%	0.326
$20,000–$49,999	30.7%	27.3%	27.3%	45.5%	
$50,000 and more	18.2%	30%	20%	50%	
Insurance					
Uninsured	5.7%	60%	0%	40%	0.321
Private	44.3%	40%	28%	32%	
Medicare	31.8%	23.5%	29.4%	47.1%	
Medicaid	13.6%	25%	62.5%	12.5	
Other	4.6%	0%	50%	50%	

Table 3 provides the baseline HbA1c mean and 95% confidence interval (CI), mean drop in HbA1c, and *p*-value for difference from baseline to 3-months follow-up for each arm. Baseline HbA1c for Group 1 was 10.0 (95% CI: 8.9, 11.0), participants dropped on average 1.25% HbA1c, which was significantly different from baseline (*p* = 0.002). Baseline HbA1c for Group 2 was 9.9 (95% CI: 9.2, 10.6), participants dropped on average 1.73% HbA1c, which was significantly different from baseline (*p* < 0.001). Baseline HbA1c for Group 3 was 10.4 (95% CI: 9.3, 11.5), participants dropped on average 1.74% HbA1c, which was significantly different from baseline (*p* < 0.001).

**Table 3:** Baseline HbA1c and 3-month Mean Drop in HbA1c by Incentive Group

Groups	N	Baseline HbA1c (95% CI)	Mean Drop in HbA1c	***P***-value
Group 1	19	10.0 (8.9, 11.0)	- 1.25%	0.002
Group 2	18	9.9 (9.2, 10.6)	- 1.73%	< 0.001
Group 3	20	10.4 (9.3, 11.5)	- 1.74%	< 0.001

## Discussion

In this pilot randomized controlled trial testing the feasibility and efficacy of structured financial incentives layered upon nurse education and skills training for African Americans with type 2 diabetes, results showed that for each of the three financial incentive groups, the magnitude in HbA1c drop from baseline to 3-month post intervention was both statistically and clinically significant. This is one of the first randomized controlled trials to test the efficacy of financial incentives layered upon nurse education and skills training among African Americans with type 2 diabetes [30].

The current findings provide preliminary evidence on the efficacy of financial incentives augmented telephone-delivered diabetes education and skills training intervention in improving metabolic control in African Americans with poorly controlled type 2 diabetes. More importantly, this study identifies financial incentives as a viable intervention strategy to motivate behavior change in African Americans with poorly controlled type 2 diabetes. The current study differs from existing literature in its use of structured financial incentives layered upon structured diabetes education and skills training. For example, a prior study compared lump financial incentives to peer mentoring among African American veterans with type 2 diabetes [23]. Specifically, participants were randomized into three groups: peer mentoring for diabetes management alone, lump financial incentive for drop in HbA1c at follow-up, or usual care. Those in the financial incentives group received a maximum of $200 if their HbA1c decreased by 2% at 6-months. If participants in the financial incentives group demonstrated a 1% drop in HbA1c at 6-months, they received $100.The study demonstrated that both peer mentoring (HbA1c drop 1.07%), and financial incentives (HbA1c drop 0.45%), were effective in reducing HbA1c at 6-months post randomization, with the greatest percent change seen in the peer group [23]. Although the study demonstrated efficacy of financial incentives in African Americans with type 2 diabetes, the absolute drop in HbA1c was small (− 0.45%). Two important limitations of this study were the use of lump incentives vs. structured incentives and the limited education and skills training provided to participants [23].

Similarly, a randomized trial conducted out of an Health Maintenance Organization (HMO) in Hawaii examined the impact of financial incentives on diabetes self-management, clinical outcomes, adopting lifestyle changes, and national recommendations for diabetes standards of medical care [27, 28]. Incentives were awarded at the time outcomes were achieved or activities completed. At 12-months, no statistically significant changes were observed across clinical outcomes (HbA1c, lipids) within or between the intervention and control groups. Additionally, no statistically significant differences were observed in standards of medical care within or between groups [27, 28]. Limitations of this study included the multiple intervention targets, the relatively small total amount of incentives at 12 months ($320) and lack of standardized educational component layered on top of the financial incentives. Additionally, the patients were primarily responsible for driving the intervention, which may be challenging for a low-income or low literacy population.

The present study integrated financial incentives into evidence-based diabetes education and skills training [5, 15, 31] across three incentive structures while providing immediate incentives for behavioral and outcome changes. By increasing frequency and timing, and by providing reminders to patients about the financial incentive as well as feedback on self-monitoring, a significant decrease was seen across financial incentive groups, suggesting this may be an important reason for the observed drop in HbA1c. The financial incentives in the current study were also structured so that incentives were large enough to motivate behavior change with rewards given for maintenance of behavior. Evidence suggests that when using financial incentives in behavioral interventions (e.g. medication adherence, weight loss and diabetes management), the role of feedback and incentive amount is a critical component to behavior change [27, 28, 32–34].

The current findings have implications across clinical practice, research, and policy. Specifically, integrating structured financial incentives into a high intensity behavioral intervention that leverages the support of nurse educators and telemonitoring demonstrates the importance of multicomponent diabetes interventions to improve outcomes in African Americans with type 2 diabetes. Components of this intervention were drawn from previous clinical trials that used cultural tailoring to provide nurse led coaching, skills training, and home telemonitoring to improve diabetes clinical outcomes among African Americans with type 2 diabetes [5, 15, 31]. Through nurse support and education, patients experience empowerment to make behavioral changes through goal setting and receive guidance through skills training [5, 15, 31]. Additionally, in light of the current restrictions as a result of the COVID-19 pandemic, use of telehealth and telephone delivered interventions have never been more critical for maintaining patient engagement. The results of the current study showing a drop in HbA1c across each financial incentive group suggests that in addition to the education, skills, and home telemonitoring, layering on financial incentives may an effective strategy in this population.

Additionally, behavioral economics is an emerging area of research that is not well tested in diabetes. Even though it is generally accepted that financial incentives can act as motivators to effect change in personal health behaviors [26], few studies have assessed the effect of financial incentives on improving metabolic outcomes in type 2 diabetes. The findings presented here have implications for the integration of behavioral economics into diabetes specific research as well as policy in the use of structured financial incentives that reward home monitoring, attendance of telephone education sessions, and lifestyle modification to lower HbA1c as a viable option for glycemic control in African Americans with type 2 diabetes.

### Limitations

This study has three key limitations that should be considered. First, given that this is a pilot study, the sample was not powered to detect between group differences and did not include a control group, however given the statistical significance observed within groups suggest the importance of financial incentives in driving behavior change. Second, as a pilot study, maintenance of behavior was not measured as a part of this intervention so these results cannot speak directly to sustainability of behavior change in the absence of financial incentives. Additionally, as all patients were aware of their involvement in the research study and the group they were assigned, it is possible that their behavior may have been influenced by this knowledge. However, the randomized controlled design of this study provides some control over such phenomenon. Finally, this study was conducted in a sample patient population located in the Southeastern United States and included only African American patients and therefore these results cannot speak to racial/ethnic differences in the use of financial incentives and may not be generalizable to all patient populations.

## Conclusion

Overall, while this trial showed significant drop in HbA1c across each incentive group, this study was limited by not being powered to detect differences between groups and as such these findings cannot speak to the effectiveness of high frequency versus moderate frequency versus low frequency incentives on glycemic control. Larger studies powered to detect the effectiveness of various incentive groups will allow us to understand which incentive structure may drive improvements in clinical outcomes for patients with diabetes. Additionally, an important unanswered question that remains is the maintenance of behavior once the incentives are removed as well as the impact of financial incentives on other clinically relevant outcomes such as quality of life. As a result of the findings of this pilot and the limitations found, the investigative team of the current study is actively examining the effectiveness of the high intensity incentive structure compared to education/skills training and home telemonitoring alone on diabetes clinical outcomes in a large randomized control trial, with sample size of 450 subjects (Clinical Trials NCT04203173). In addition, this new trial is also examining within racial/ethnic group differences and socioeconomic differences in efficacy of high intensity financial incentives to determine if there is differential efficacy within groups. Finally, we will examine maintenance of behavior by removing the incentives and following patients for an additional recommended 6 months to understand if behavior change is sustained without further incentives [35–40].

## Data Availability

Data used for this study is available upon reasonable request from LEE.

## Abbreviations

HbA1c: Hemoglobin A1c
IRB: Institutional Review Board
CI: Confidence Interval
HMO: Health Maintenance Organization
